# The combination of everolimus and zoledronic acid increase the efficacy of gemcitabine in a mouse model of pancreatic adenocarcinoma

**DOI:** 10.18632/oncotarget.25560

**Published:** 2018-06-15

**Authors:** Carole Vitellius, Lionel Fizanne, Elodie Menager-Tabourel, Joelle Nader, Nathalie Baize, Margot Laly, Emilie Lermite, Sandrine Bertrais, FX Caroli-Bosc

**Affiliations:** ^1^ Department of Gastroenterology, University Hospital Centre, Angers, France; ^2^ HIFIH, Laboratory, UNIV Angers, Université Bretagne Loire, Angers, France; ^3^ Department of Digestive Surgery, University Hospital Centre, Angers, France

**Keywords:** everolimus, zoledronic acid, gemcitabine, pancreatic cancer

## Abstract

**Background:**

Gemcitabine is a standard treatment for pancreatic adenocarcinoma. Many mechanisms are involved in gemcitabine resistance, such as reduced expression of the human equilibrative nucleoside transporter 1 (hENT1) membrane transporter, deoxycytidine kinase deficiency, and changes in the signal transmission of mitogen-activity protein kinase (MAPK) and the phosphoinositide 3-kinase (PI3K) pathways.

**Aim:**

To evaluate the anti-tumor efficiency of blocking signaling pathways using combined action of gemcitabine, everolimus and zoledronic acid versus gemcitabine alone in a mouse subcutaneous xenograft.

**Methods:**

Implantations of two human pancreatic adenocarcinoma cells lines (PANC1, K-ras mutated and gemcitabine-resistant; and BxPc3, wild-type K-ras and gemcitabine-sensitive) were performed on male athymic nude mice. The mice received different treatments: gemcitabine, gemcitabine plus everolimus, everolimus, gemcitabine plus zoledronic acid, everolimus plus zoledronic acid, or gemcitabine plus everolimus and zoledronic acid, for 28 days. We measured the tumor volume and researched the expression of the biomarkers involved in the signaling pathways or in gemcitabine resistance.

**Results:**

In wild-type K-ras tumors, the combinations of gemcitabine plus everolimus; zoledronic acid plus everolimus; and gemcitabine plus zoledronic acid and everolimus slowed tumor growth, probably due to caspase-3 overexpression and reduced Annexin II expression. In mutated K-ras tumors, gemcitabine plus everolimus and zoledronic acid, and the combination of zoledronic acid and everolimus, decreased tumor volume as compared to gemcitabine alone, inhibiting the ERK feedback loop induced by everolimus.

**Conclusion:**

The combination of zoledronic acid and everolimus has an antitumor effect and could increase gemcitabine efficacy.

## INTRODUCTION

Pancreatic ductal adenocarcinoma (PDAC) is becoming the second leading cause of cancer-related deaths in France [1], and fourth in the world [2]. Moreover, it is the digestive cancer with the worst prognosis, with a 5-year overall survival rate of 7%, as found in the EUROCARE study [3]. Gemcitabine (2'-2'-difluorodeoxycytidine) is a standard chemotherapy treatment for all stages of pancreatic adenocarcinoma. However, the survival benefit and clinical impact remain modest due to the high degree of intrinsic and acquired resistance. In the last decade, many randomized trials involving gemcitabine combinations were performed on metastatic patients, however they failed to demonstrate a statistically significant survival advantage over gemcitabine alone, with the exception of combinations with erlotinib, which provided few benefits [4], and more recently with nab-paclitaxel [5]. However, the interest of using gemcitabine in the treatment of PDAC is still relevant. Theoretically, it remains the standard treatment for patients over the age of 75 and patients whose WHO status is less than or equal to 2. In an adjuvant situation, it currently represents the reference treatment in combination with capecitabine [6]. Gemcitabine is a pyrimidine analog transformed by deoxycytidine kinase (dCK) into gemcitabine diphosphate (dFdCTP) and gemcitabine triphosphate (dFdCTP). DNA incorporation of these metabolites inhibits DNA synthesis and induces apoptosis. The induction of apoptosis through caspase signaling is an important action mechanism [7]. Gemcitabine resistance mechanisms are complex, involving both the tumor microenvironment and the intrinsic resistance [8]. Gemcitabine resistance seems to be partly related to changes in gene expression involved in gemcitabine transport and metabolism. Two main types of resistance were described: reduced expression of human equilibrative nucleoside transporter-1 (hENT-1) and deficiency in dCK activity, which plays a major role in the cellular transformation of gemcitabine into active metabolites [8]. Other mechanisms are also involved in gemcitabine resistance, such as Annexin a2 overexpression through activation of the Akt/mTOR apoptotic pathway [8, 9]. It has been shown that everolimus has an additive antiproliferative effect in gemcitabine-treated pancreatic tumor cells *in vitro* [10]. Otherwise, PDAC is the cancer with the highest K-ras mutation frequency (>90%) [11]. The MAPK pathway, particularly K-ras activating mutations, plays a major role in pancreatic carcinogenesis [12]. Ras proteins alternate between GTP-bound that represents the “On state” and GDP-bound that was the “Off state”. The transition between these two states requires proteins accelerating GTP hydrolysis, GTPase activating proteins (GAPs). Mutated K-ras prevents the intrinsic and GAPs catalyzed hydrolysis of GTP, thereby generating permanently active RAS and constitutive activation of cell proliferative signals [13]. This mutation leads to a reaction cascade marked by the successive activation of proteins involved in the MAPK pathway: Raf, MEK and ERK. It has also been suggested that pancreatic carcinogenesis could strongly rely on the dysregulated activity of the p21ras. Indeed, it has been shown that zoledronic acid has antiproliferative effects on the p21ras/Raf1/MEK/ERK1-2 mitogenic pathway and Pkb/Akt survival signaling through ERK inhibition. Zoledronic acid also induces apoptotic death of human pancreatic cancer cells *in vitro* [14]. Conversely, it has been shown that K-Ras mutation induced feedback ERK activation contributes to the rapalog resistance in pancreatic ductal adenocarcinomas [15] and that PI3K pathway activation mediates resistance to MEK inhibitors in K-ras mutant cancers [16]. It is therefore not surprising that everolimus used as a single agent does not have an anti-tumor effect in patients with gemcitabine-refractory metastatic PDAC [17]. We hypothesized that simultaneous blocking of both signaling pathways using everolimus and zoledronic acid could increase the efficacy of gemcitabine.

## RESULTS

Eighty-five mice were included in the study, divided according to Table 1.

**Table 1:** Groups of treatment

BxPc3 n=59	P n=8	G n=9	G + E +AZ n=10	E+AZ n=10	G+AZ n=7	G+E n=8	E n=7
PANC-1 n=26	P n=8	G n=5	G + E + AZ n=7	E + AZ n=6			

Mices were separated depending on Xenograft of cells lines BxPc3 and PANC-1 and they received different combination of treatments.

Placebo (P), Gemcitabin (G), Gemcitabin + Everolimus + Zoledronic acid (G+E+AZ), Gemcitabin + Everolimus (G+E), Gemcitabin + Zoledronic acid (G + AZ), Everolimus + Zoledronic acid (E+AZ) and Everolimus (E).

The initial tumor volume was 113.1 +/-24.4 mm^3^ in the BxPc3 group, compared to 108.9 +/-20.7 mm^3^ in the PANC1 group. There was no significant difference between groups according to the non-parametric Mann-Whitney-Wilcoxon test (p=0.60). Moreover, there was no significant difference between the weights of the mice in the different groups (Mann-Whitney-Wilcoxon, p=0,06).

### Weight variation

The weight variation was no different between the BxPc3 and PANC-1 groups.

### Tumor volume

The mean change in tumor volume was 450.9 +/- 189.3 mm^3^ for the placebo, 265.1 +/-126.9 mm^3^ for gemcitabine, 76.1 +/-56.2 for G+E+AZ and 78.0 +/-52.8 for E+AZ in BxPc3 group.

The mean change in tumor volume was 123.9 +/- 124.3 mm^3^ for the placebo, 75.0 +/-23.1 mm^3^ for gemcitabine, -40.4 +/-34.8 for G+E+AZ and -44.8 +/- 19.4 for E+AZ in PANC-1 group. The tumor volume change was significantly higher in BxPc3 than in PANC-1: placebo p=0.011; gemcitabine p=0.041; E+AZ p=0.006 and G+E+AZ p=0.006.

In the placebo and gemcitabine groups, the tumor volume increased, however this increase was smaller in the PANC-1 group. For E+AZ and G+E+AZ, the tumor volume increased slightly in the BxPc3 group, whereas it decreased in the PANC-1 group. These differences were always significant after weight variation adjustment.

### BxPc3 group

**Figure 1:**
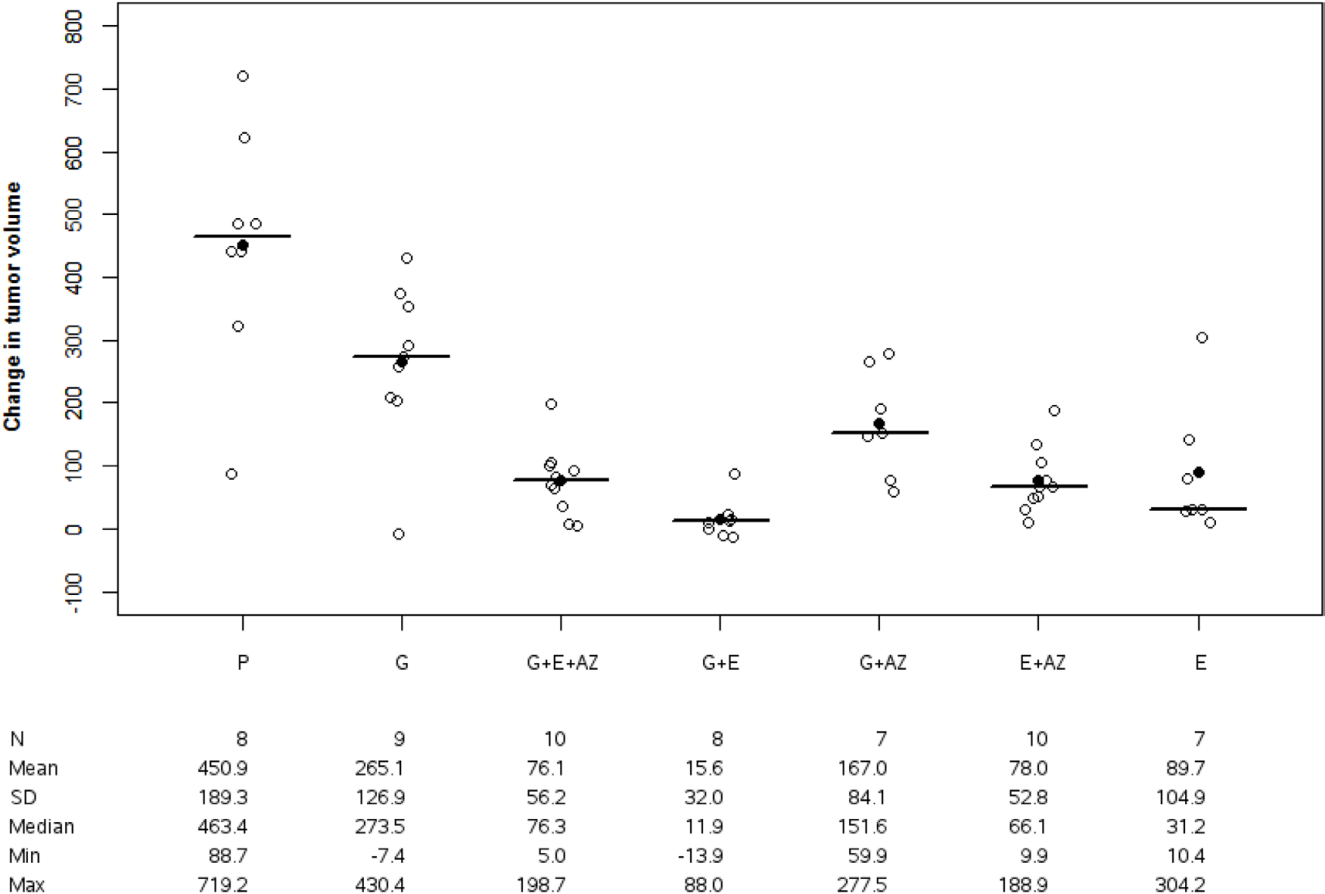
Tumor volume change in BxPc3 Tumor volume change was significantly decreased with G+E+AZ, G+E in BxPc3 mices. Placebo (P), Gemcitabin (G), Gemcitabin + Everolimus + Zoledronic acid (G+E+AZ), Gemcitabin + Everolimus (G+E), Gemcitabin + Zoledronic acid (G + AZ), Everolimus + Zoledronic acid (E+AZ) and Everolimus (E). SD: standard deviation.

The tumor volume variation was significantly higher in the placebo group versus gemcitabine (p=0.032) G+E+AZ (p=0.006), G+E (p=0.005), G+AZ (p=0.021) versus E+AZ (p=0.005) and E (p=0.011). This increase was significantly lower for G+E+AZ (p=0.011), G+E (p=0.012) and E+AZ (p=0.011) as compared to the gemcitabine group. The best tumor response was observed in G+E (Figure 1).

### PANC1 group

**Figure 2:**
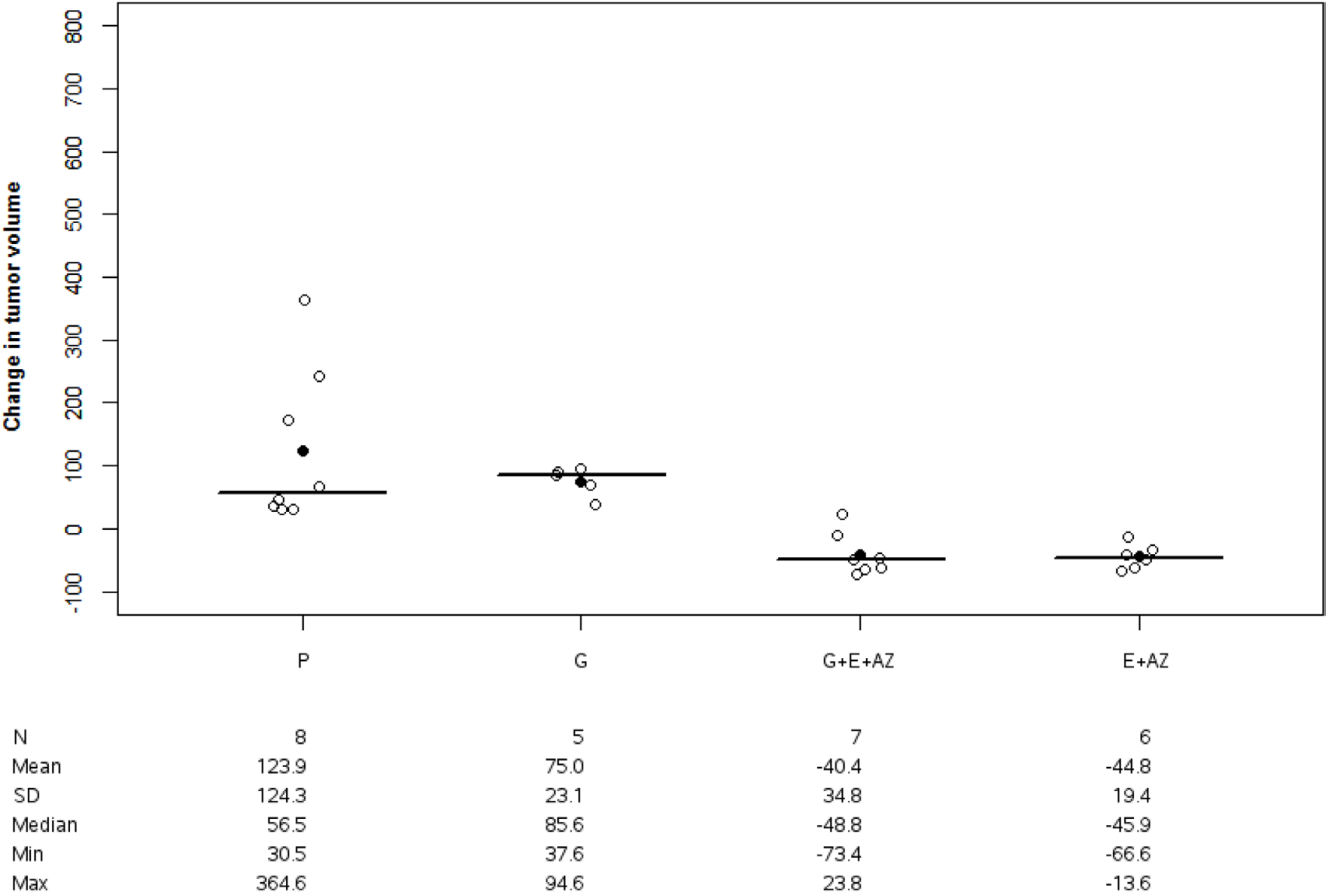
Tumor volume change in PANC1 Tumor volume change was significantly decreased with G+E+AZ and E+ AZ in PANC-1 mices. Placebo (P), Gemcitabin (G), Gemcitabin + Everolimus + Zoledronic acid (G+E+AZ) and Everolimus + Zoledronic acid (E + AZ). SD: standard deviation.

There was no significant tumor volume variation between the placebo and gemcitabine groups (p=0.721). These variations significantly decreased for G+E+AZ (p<0,01) and E+AZ (p<0,01) as compared to placebo and gemcitabine (p=0.019 for G+E+AZ and p=0.024 for E+AZ) (Figure 2).

### Signaling pathways

#### Ras/Raf/MAPK

#### BxPc3 versus PANC-1 group

In BxPc3, the ERK-p expression was 86.9 +/-31.2% in placebo, 120.3 +/- 27.6 % in gemcitabine, 160.2 +/- 55.4% in E + AZ and 134.3 +/- 48.5% in G + E + AZ. In PANC-1, the ERK-p expression was 111.8 +/- 65.9 in placebo, 54.0 +/- 7.3 % in gemcitabine, 142.4 +/-56.0 in E + AZ and 69.8 +/- 15.1 in G + E + AZ. There was an higher expression of ERK-p in gemcitabine BxPc3 group than in gemcitabine PANC-1 group (p=0.008) and in G+ E+ AZ in BxPc3 versus in G + E + AZ in PANC-1 (p=0.043).

#### BxPc3 group

There was no significant difference in Raf1 tumor expression between groups (p=0.112) (Table 2).

**Table 2:** ERK-p, RAF-1, AKT, mTOR, Ki67, Annexin II, Caspase 3, hENT1 expression in BxPC3

n=59	P n=8	G n=9	G + E + AZ n=10	E + AZ n=10	G + AZ n=7	G + E n=8	E n=7	p
ERK-p n Me +/-SD Median Min Max	8 86.9 +/-31.2 77.1 55.85 147.56	7 120.3 +/-27.6 122.9 74.86 153.53	10 134.3 +/- 48.5 130.3 70.02 226.90	9 160.2 +/-55.4 142.6 76.99 250.56	7 143.4 +/-68.3 140.7 67.42 283.04	7 172.0 +/- 42.7 160.3 124.64 235.76	5 134.5 +/- 29.6 146.0 98.08 165.52	<0.018
RAF-1 n Mean +/- SD Median Min Max	6 99.7 +/-34.9 81.6 72.59 148.36	9 79.4 +/-27.5 70.9 44.93 129.7	6 97.3 +/- 40.4 106.5 39.21 143.40	6 130.1 +/- 38.9 130.3 70.73 184.97	7 120.7 +/- 24.3 117.3 95.33 168.47	8 124.8+/-38.2 120.2 68.4 176.2	7 110.2 +/-39.7 92.6 61.8 170.8	0.112
AKT n Mean +/- SD Median Min Max	8 81.9 +/- 15.0 81.2 61.87 102.53	8 87.8 +/- 16.5 86.6 68.17 113.54	10 89.4 +/- 19.4 91.4 52.65 129.03	9 108.4 +/- 30.0 103.0 70.92 154.94				0.209
mTOR n Mean +/- SD Median Min Max	7 86.6 +/- 26.5 86.6 45.53 133.13	8 116.0 +/- 71.3 117.8 10.20 237.14	8 75.5 +/- 36.3 69.0 21.33 148.08	6 57.6 +/- 40.1 48.9 19.13 129.19	4 124.3 +/- 42.0 120.2 84.95 171.92	7 91.4 +/- 43.3 75.1 46.21 172.94	7 123.6 +/-41.5 116.3 56.24 193.18	0.090
Ki67 n Mean +/- SD Median Min Max	8 49.0 +/- 9.0 48.3 32.15 60.69	8 41.9 +/- 13.6 46.6 20.07 62.10	10 46.6 +/- 8.4 47.3 35.81 59.91	7 55.3 +/- 7.9 51.5 48.85 68.80	6 53.2 +/- 11.4 58.3 34.09 62.92	7 53.9 +/- 9.3 51.9 43.29 66.58	6 55.7 +/- 7.2 55.5 48.38 65.23	0.140
Annexin II n Mean +/- SD Median Min Max	8 216.3 +/- 135.4 169.2 100.95 428.34	8 340.9 +/-53.5 334.6 251.52 424.04	8 168.5 +/- 29.6 183.7 115.09 200.66	8 221.6 +/- 41.6 208.0 178.88 297.35	4 78.0 +/- 22.8 81.6 50.44 98.46	7 107.0 +/- 36.7 119.9 50.37 148.47	6 146.7 +/- 98.7 146.4 32.73 275.40	<0.001
Caspase 3 n Mean +/- SD Median Min Max	7 69.8 +/- 27.3 72.6 40.40 109.98	7 87.6 +/- 64.4 69.2 -2.65 175.39	7 89.3 +/- 34.7 89.0 40.26 129.69	8 93.0 +/- 68.2 98.4 0.13 215.15	7 138.9 +/-28.9 125.5 106.4 178.4	5 136.9 +/- 47.9 123.6 86.76 216.14	6 151.6 +/- 57.0 136.0 89.80 234.64	0.023
hENT1 n Mean +/- SD Median Min Max	8 140.6 +/- 40.2 133.6 83.68 192.98	8 167.3 +/- 59.8 153.8 106.19 306.22	8 177.3 +/- 68.6 168.1 107.81 334.14	7 198.2 +/- 67.5 200.2 109.78 297.35	7 116.3 +/-36.4 105.8 81.7 170.8	8 112.9 +/-24.3 111.9 83.8 145.8	7 85.1 +/-20.8 93.9 40.63 100.16	<0.001

Placebo (P), Gemcitabin (G), Gemcitabin + Everolimus + Zoledronic acid (G+E+AZ) and Everolimus + Zoledronic acid (E + AZ).

SD: standard deviation.

**Figure 3:**
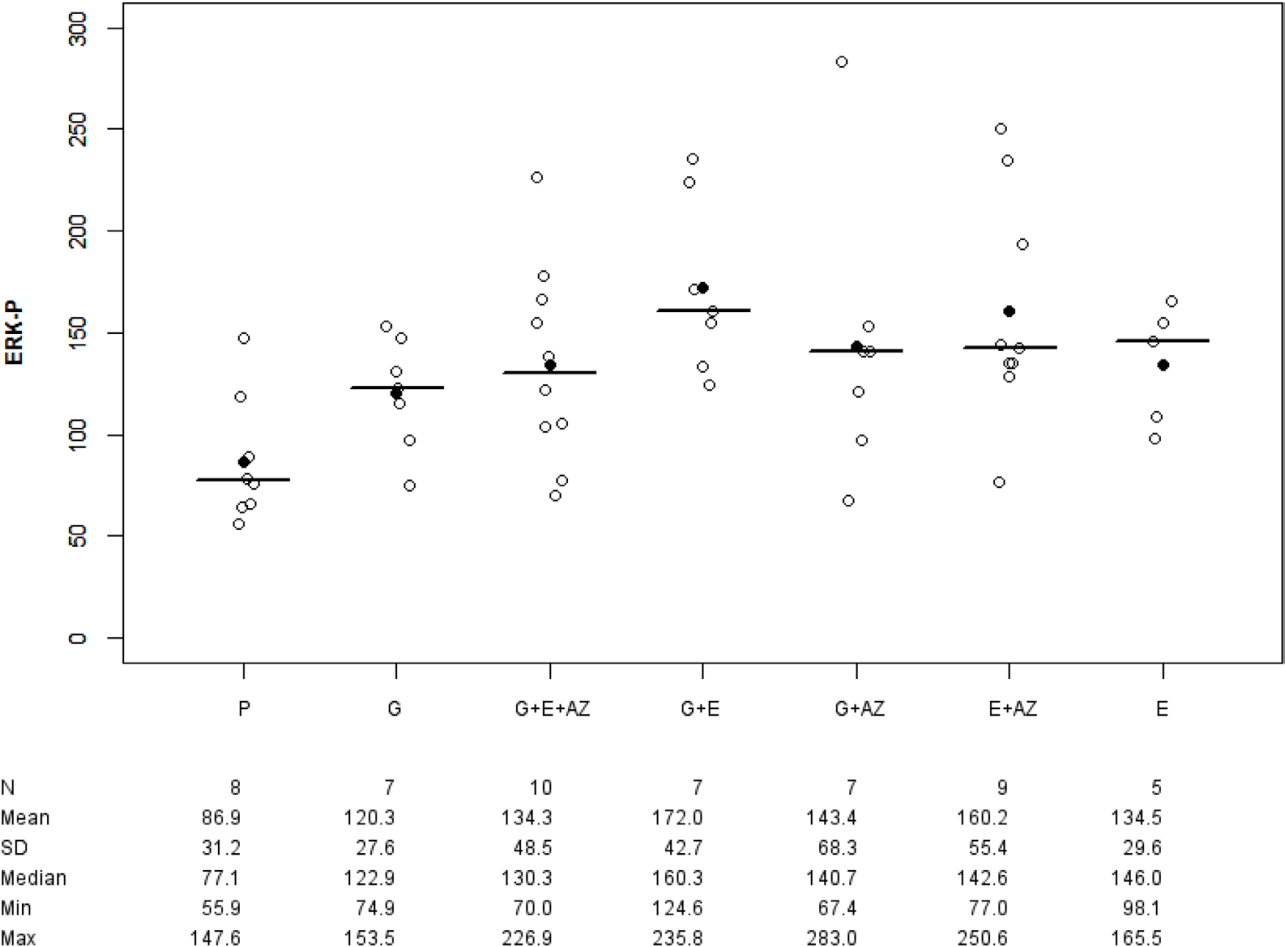
ERK-p expression in BxPc3 Placebo (P), Gemcitabin (G), Gemcitabin + Everolimus + Zoledronic acid (G+E+AZ), Gemcitabin + Everolimus (G+E), Everolimus + Zoledronic acid (E+AZ) and Everolimus (E). SD: standard deviation.

There was an overexpression of ERK-p for E (p=0.028), G+E (p=0.003), E+AZ (p=0.009), G + AZ (p=0.037) and G + E + AZ (p=0.026) as compared to the placebo (Figure 3). And there was an overexpression of ERK-p for G+ E as compared to gemcitabine (p=0.016).

There was no significant difference in Ki67 tumor expression between groups (p=0.140).

#### PANC-1 group

Only ERK-p tumor expression was studied.

**Figure 4:**
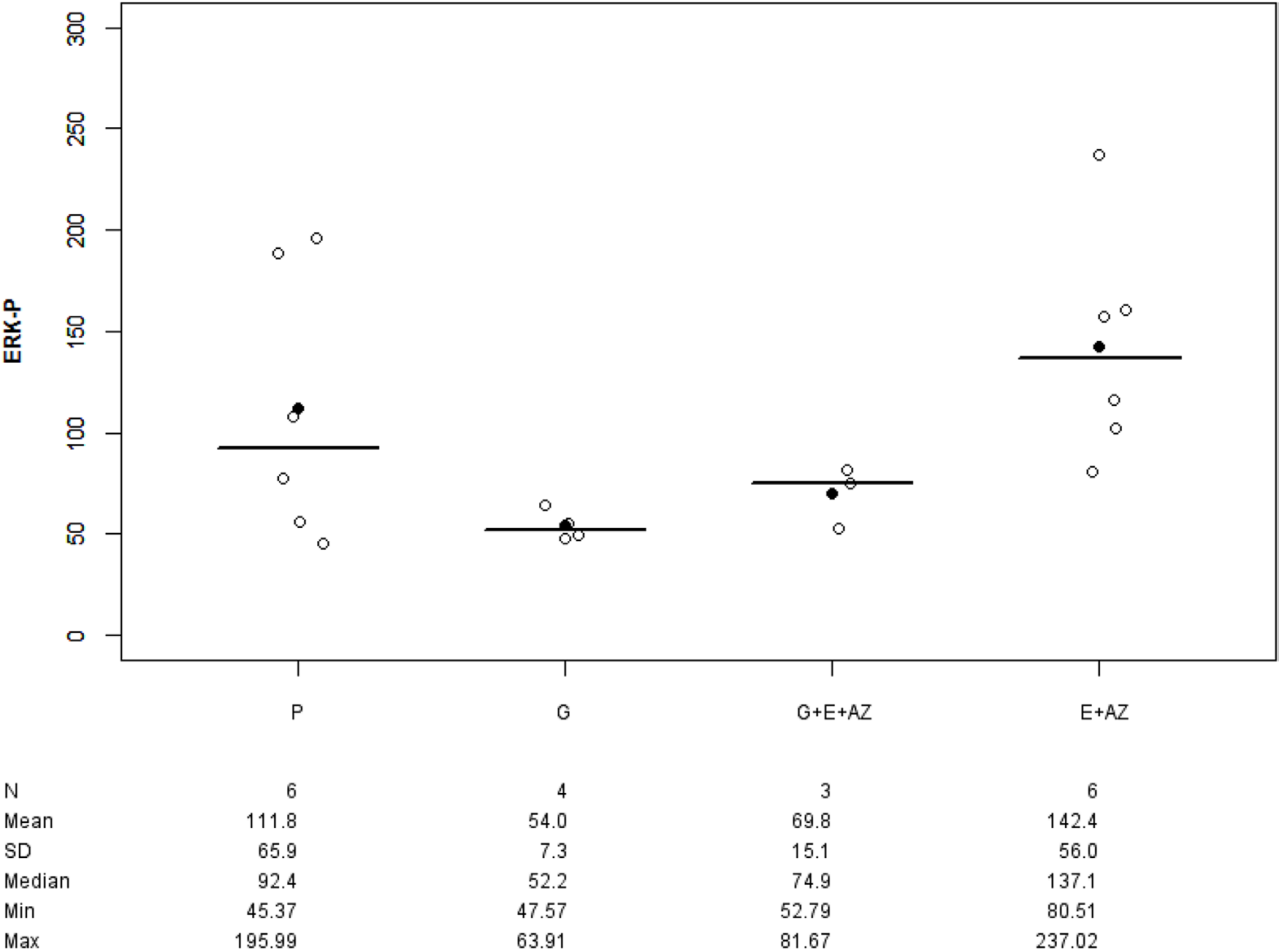
ERK-p expression in PANC-1 Placebo (P), Gemcitabin (G), Gemcitabin + Everolimus + Zoledronic acid (G+E+AZ), Everolimus + Zoledronic acid (E+AZ). SD: standard deviation.

There was no significant difference in groups as compared to the placebo. The ERK-p tumor expression significantly increased for E+AZ as compared to gemcitabine (p=0.011) (Figure 4).

### PI3K/AKT/MTOR

#### BxPc3 versus PANC-1 group

There was a lower expression of AKT in placebo BxPc3 versus placebo PANC-1 (p=0.016) (Table 2). There was no difference between the others groups.

#### BxPc3 group

There was no significant difference in tumor expression of AKT between groups (p=0.209) (Table 2).

There was no significant difference in tumor expression of mTOR between groups (p=0.90) (Table 2).

**Figure 5:**
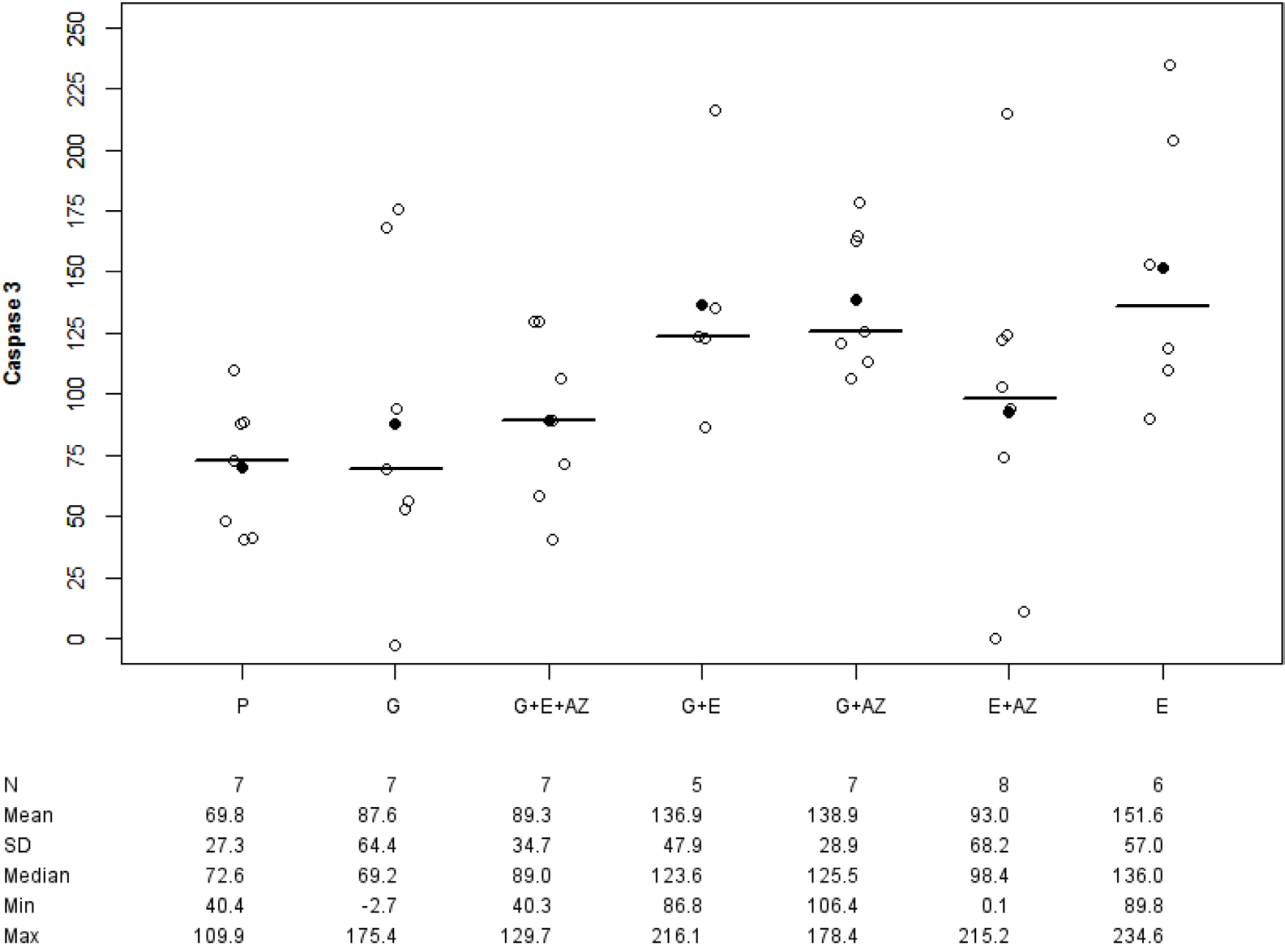
Caspase-3 expression in BxPc3 Placebo (P), Gemcitabin (G), Gemcitabin + Everolimus + Zoledronic acid (G+E+AZ), Gemcitabin + Everolimus (G+E), Everolimus + Zoledronic acid (E+AZ) and Everolimus (E). SD: standard deviation.

There was an overexpression of Caspase-3 tumor in G+ E (p= 0.019), G + AZ (p= 0.003) and E (p= 0.007) versus placebo (Figure 5).

#### PANC-1 group

There was no significant difference in tumor expression of AKT between groups (p=0. 057) (Table 3).

**Table 3:** ERK-p, AKT, hENT1 and Annexin II expression in PANC-1 group

n=26	P n=8	G n=5	G + E + AZ n=7	E + AZ n=6	p
ERK-p n Mean +/-DS Median Min Max	6 111.8 +/- 65.9 92.4 45.37 195.99	4 54.0 +/-7.3 52.2 47.57 63.91	3 69.8 +/- 15.1 74.9 52.79 81.67	6 142.4 +/- 56.0 137.1 80.51 237.02	0.040
AKT n Mean +/-DS Median Min Max	8 145.7 +/- 67.8 136.9 60.89 286.53	4 69.2 +/- 25.6 74.8 33.63 93.50	3 98.6 +/- 31.6 80.7 80.05 135.01	6 119.9 +/- 16.5 127.2 92.27 134.36	0.057
hENT1 n Mean +/-DS Median Min Max	8 79.6 +/-22.1 87.4 38.39 102.17	4 87.7 +/- 13.4 91.4 69.35 98.91	4 126.6 +/- 33.6 120.5 95.69 169.84	6 117.7 +/- 29.5 124.3 78.58 159.20	0.037
Annexin II n Mean +/-DS Median Min Max	8 91.4 +/- 40.9 90.4 45.86 137.89	5 119.6 +/- 13.8 125.5 97.97 130.76	4 81.7 +/- 10.4 83.5 69.38 90.32	6 96.2 +/- 36.2 105.2 32.64 128.78	0.261

Placebo (P), Gemcitabin (G), Gemcitabin + Everolimus + Zoledronic acid (G+E+AZ) and Everolimus + Zoledronic acid (E + AZ)

SD: standard deviation.

### Mechanisms of resistance to gemcitabine

The hENT1 expression in placebo groups was 140.6 +/- 40.2 % in BxPc3 and 79.6 +/- 22.1 % in PANC-1. In gemcitabine groups, this expression was 167.3 +/- 59.8% in BxPc3 and 87.7 +/- 13.4% in PANC-1. The hENT1 expression in E + AZ was 198.2 +/-675% in BxPc3 and 117.7 +/- 29.5% in PANC-1. In G + E +AZ this expression was 177.3 +/- 68.6% in BxPc3 and 126.6 +/-33.6% in PANC-1. There was a significant decrease in hENT1 expression for the placebo (p=0.06), gemcitabine (p=0.007) and E + AZ (p=0.032) in the PANC-1 group as compared to BxPc3. These differences were not significant for G+E+AZ (p=0.126).

In BxPC3, the Annexin II expression was 216.3 +/-135.4% in placebo, 340.9 +/-53.5 % in gemcitabine, 221.6 +/-41.6% in E + AZ and 168.5 +/-29.6 % in G + E + AZ. In PANC-1, The Annexin II expression was 91.4 +/- 40.9% in placebo, 119.6 +/- 13.8% in gemcitabine, 96.2 +/- 36.2% in E + AZ and 81.7 +/- 10.4% in G + E +AZ. There was a significant lower in Annexin II expression in placebo (p=0.036), gemcitabine (p=0.003), E+AZ (p=0.002) and G+E+AZ (p=0.007) in PANC-1 group as compared to BxPc3.

### BxPc3 group

There was a significant decrease in tumor expression of hENT1 in everolimus versus placebo (p=0. 008, Table 2). And there was a significant decrease of hENT1 in E (p=0.001) and G+ E (p=0.006) versus gemcitabine (data not shown).

**Figure 6:**
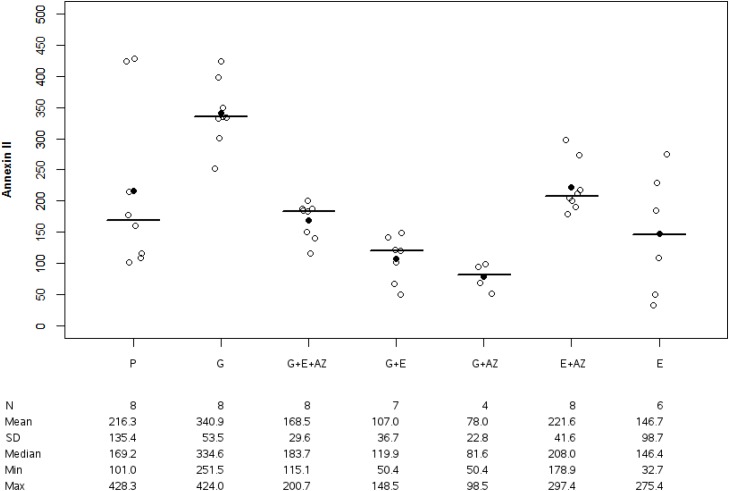
Annexin II expression in BxPc3 Placebo (P), Gemcitabin (G), Gemcitabin + Everolimus + Zoledronic acid (G+E+AZ), Gemcitabin + Everolimus (G+E), Gemcitabin + Zoledronic acid (G + AZ), Everolimus + Zoledronic acid (E+AZ) and Everolimus (E). SD: standard deviation.

Annexin II expression was significantly lower for G + AZ versus placebo (p=0.007) and in G+E+AZ (p=0.001), G+E (p=0.001), G+AZ (p=0.007), E+AZ (p=0.002) and E (p=0.003) as compared to the gemcitabine group (Figure 6).

### PANC-1 group

**Figure 7:**
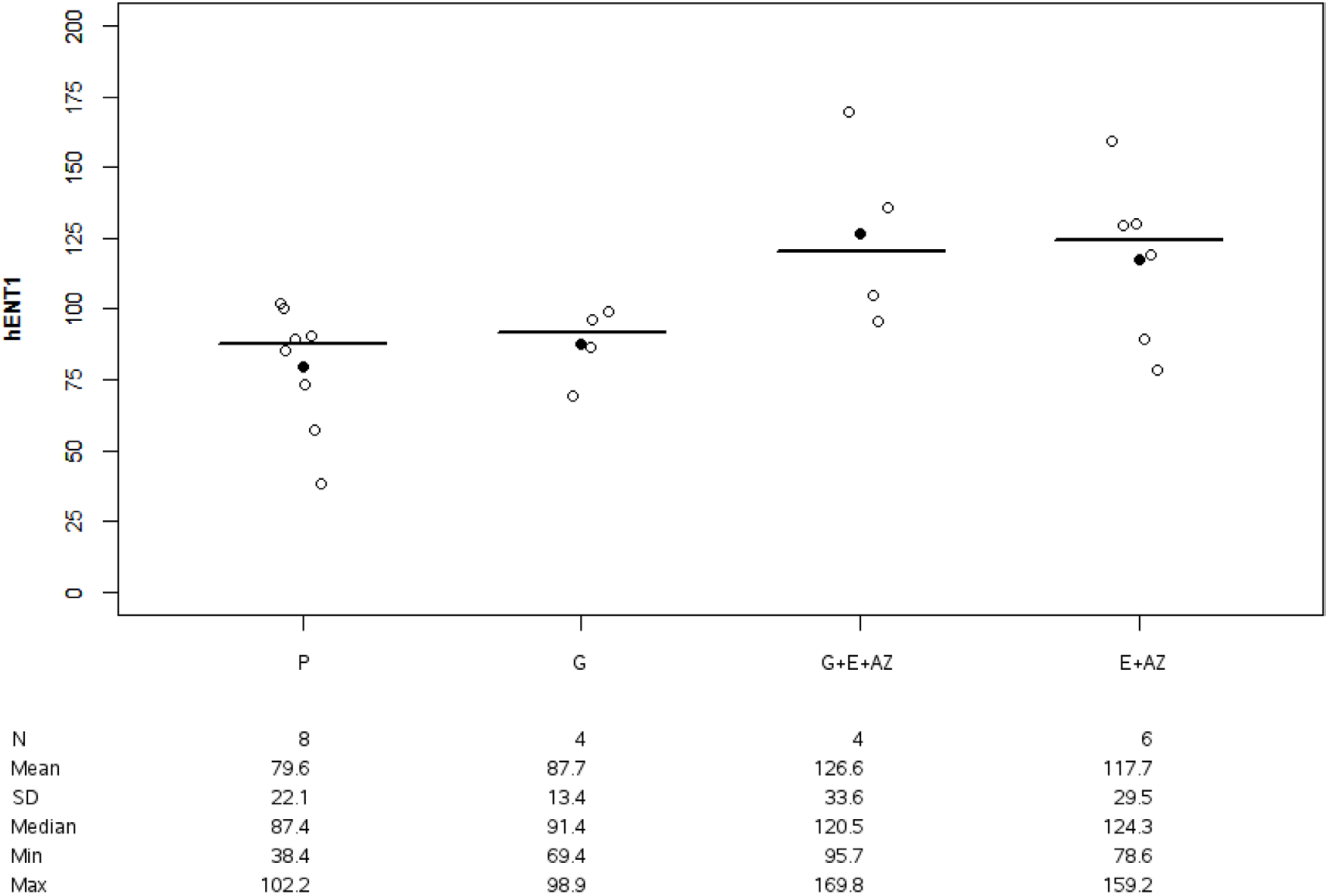
HENT1 expression in PANC1 Placebo (P), Gemcitabin (G), Gemcitabin + Everolimus + Zoledronic acid (G+E+AZ) and Everolimus + Zoledronic acid (E + AZ). SD: standard deviation.

HENT1 expression was significantly higher for G+E+AZ (p=0.017) and E + AZ (p=0.039) as compared to the placebo (Figure 7). There was no significant difference between G+E+AZ (p=0.083) and E+AZ (p=0.136) as compared to the gemcitabine group.

There was no significant difference in tumor expression of Annexin II between the groups (p=0. 261).

### Tolerance

All mice were alive at the end of the study.

For BxPc3, there was a significant reduction in weight between G+E (p=0.017) and G+E+AZ (p=0.031) as compared to the placebo group, and there was a significant reduction in weight between G+E (p=0.017) and G+E+AZ (p=0.013) as compared to the everolimus group (data not shown). There was a significant reduction of weight for G+E (p=0.036) as compared to the gemcitabine group.

For PANC-1 there was no significant variation in weight of mices.

## DISCUSSION

Gemcitabine has been shown to demonstrate significant clinical activity against pancreatic adenocarcinoma [18]. It is therefore important to effectively target patients who respond to gemcitabine and to identify biomarkers that may interfere with its metabolism in order to increase its anti-tumor efficacy. Our study focused on blocking the signaling pathways involved in pancreatic adenocarcinoma. Our hypothesis was that simultaneous blocking of the mTOR and MAPK pathways upstream ERK pathway could increase gemcitabine's anti-tumor effect. MTOR exists in two distinct functional complexes: mTOR complex 1 (mTORC1) and mTOR complex 2 (mTORC2). The mTORC1 pathway is also activated by the frequent K-ras mutation in pancreatic cancer and drives cancer progression [19]. It is regulated through two negative feedback loops, which activate PI3K and ERK in pancreatic cancer, resulting in the cancer's resistance to the treatment [20–22]. To test our hypothesis, we used a model of orthotopic xenograft in nude mice from human tumor cell lines, either gemcitabine-sensitive (Wt K-ras, BxPc3 mice) or gemcitabine-resistant (mt-K-ras PANC-1 mice). We chose to block the Akt/mTOR pathway at the mTORC1 receptor using everolimus and to block the MAPK pathway at p21 ras using zoledronic acid, as previously suggested [14]. Almost 90% of pancreatic cancers are K-ras mutated [11]. We had first tested our treatments in wild-type K-RAS cells, sensitive gemcitabine (BxPc3 arm) to select the more efficient combined associations. In our study the associations of gemcitabine plus everolimus, everolimus plus zoledronic acid and gemcitabine plus everolimus and zoledronic acid were more efficient than gemcitabine alone. In the literature, studies have suggested that everolimus used alone inhibits the tumor growth of BxPc3 (wt RAS) cells, but could not block tumor growth in PANC1 (mutated K-ras) mice because everolimus causes an ERK feedback loop and is therefore opposed to the anti-tumor action [15]. So for PANC1 only combinations with everolimus and zoledronic acid were studied. In our study, tumor volume and weight of mice were similar at the initial time of the experimentation, regardless of the group of mice. No toxicity of the different combinations was observed in previous analyses, especially renal failure (data not shown). As in BxPc3 group, the combination of everolimus, zoledronic acid and gemcitabine or zoledronic acid and everolimus are more efficient than gemcitabine alone in mt K-ras tumors. Interestingly, tumor growth decreased in gemcitabine-resistant tumors, whereas it only slowed in gemcitabine-sensitive tumors. In sensitivity tumors, growth is slowed down by gemcitabine alone and this effect is accentuated by the addition of everolimus but not by the addition of zoledronic acid whereas in mutated tumors, the addition of zoledronic acid to everolimus decreases tumor growth. The addition of zoledronic acid could play a role through its interaction with p21ras/raf-1/MEK1/ERK [14].

The mechanisms implied in response of treatments seem to be different depending on cells lines (BxPc3 and PANC-1). Interestingly, it has been suggested that the everolimus-induced PI3K feedback loop does not contribute directly to the rapalog resistance in K-ras-mutated PDAC cells [15]. In our study, an overexpression of ERK-p was found for G+E in BxPc3 and for E+AZ in PANC-1, confirming that everolimus activate ERK without impact on efficacy of treatment. The predominant anti-tumor mechanism in gemcitabine-sensitive tumors appears to be associated with apoptosis, as suggested by caspase-3 overexpression. Constitutive activation of nuclear factor κB (NF-κB) is frequently observed in PDAC [23], which suggests that for mt K-ras tumors, zoledronic acid could reduce mTOR resistance and thus enhance the therapeutic efficacy of everolimus, perhaps by inhibiting NF-κB, as previously shown in breast tumors [24]. Otherwise, in addition, a recent study reported disappointing results of a double inhibition of PI3K and MEK in a second-line treatment following the failure of gemcitabine [25]. This seems to reinforce the idea that the blocking of passing bridges between the two signaling pathways plays a key role. Considered together, these results indicate the zoledronic acid in association with gemcitabine could block the everolimus-induced ERK feedback loop and thus enhance the therapeutic efficacy of everolimus in treating mt K-ras PDAC and could sensitize the mutated K-ras tumors to chemotherapy. Understanding the pathways involved in PDAC seems insufficient to explain the efficiency of these therapeutics. Moreover, Zhao et al. have shown that the anti-tumor effect of zoledronic acid was mediated partly by the inhibition of angiogenesis [26]. Annexin II seems to be an important factor of gemcitabine resistance through the interruption of the Akt/mTOR pathway [9]. The results of our study coincide with those of Zhao et al., and the expression of Annexin II was significantly under-expressed in the arms treated with everolimus, zoledronic acid and the combination in the BxPc3 group, with an overexpression of caspase-3. The expression of Annexin II is decreased in all groups of PANC-1 as compared to BxPc3, but this expression was not different between PANC-1 groups, so this mechanism seems not to be implied in efficacy of treatments in mt K-ras tumors. Our work confirms the results of a previous study suggesting that Annexin II overexpression is a factor of poor response to gemcitabine [27], especially in wt K-ras tumors. Consequently, a decrease in Annexin II activity would increase response to gemcitabine, perhaps by modulating the activity of NFKB [28]. Annexin II does not play a role in mt K-ras tumors. Reduction of hENT1 expression is an other resistance factor to gemcitabine [8, 29] and patients with a higher expression of hENT1 seem to have a longer survival. In our study, the impact of hENT1 is not clear because its expression was decreased in gemcitabine plus everolimus and everolimus as compared to placebo or gemcitabine in wt K-RAS tumors without impact of efficacy. In gemcitabine-resistant tumors, hENT1 expression was higher in gemcitabine plus everolimus and zoledronic acid and in everolimus plus zoledronic acid as compared to placebo without significant difference in comparison to gemcitabine maybe by a lack of power. That explains probably not the antitumoral action of these treatments because everolimus and zoledronic acid have not an action on and by hENT1. Their efficacy is probably due to a specific mechanism induced by everolimus and zoledronic acid combination, unknown at this time. This may explain the negativity of II studies evaluating the action of everolimus in K-ras-possibly mutated tumors [17, 30].

Our study shows that the combination of everolimus and zoledronic acid appears to be effective in adenocarcinoma pancreatic with a best response in mutated K-ras tumors. The mechanisms implied in efficacy of treatments seem to be different according to gemcitabine sensitivity and K-ras mutation. The decreased expression of Annexin II and apoptosis seem to be important in wt K-ras tumors. The mechanisms involved were not fully understood and further clinical trials are necessary. The findings may be relevant for the use of these products in future trials.

## MATERIALS AND METHODS

### Animals

All experiments involving animals were conducted in accordance with European regulations on the protection of animals used for scientific purposes (Directive 2010/63/EU). The Ethics Committee of Animal Experimentation of Pays de la Loire (No approved the protocol. CEEA.2011.27).

Eight-week old male athymic nude mice (BALB/cAnNRJ –Foxn1nu/Foxn1nu) were obtained from Janvier laboratories (Saint-Berthevin, France). The mice were kept under sterile conditions in a humidity and temperature-controlled room with 12-hour alternations of light and darkness, at the animal facility of Angers University Hospital (SCAHU, *Service commun d’animalerie hospitalo-universitaire*). They had free access to food and water.

### Cell lines

Human pancreatic cancer cell lines, PANC-1 and BxPC-3, were purchased from LGC Standards, a partner of the American Type Culture Collection (USA). The PANC-1 cell line was cultured in DMEM and BxPC-3 in RPMI-1640; with 10% fetal bovine serum, 1% glutamine, 1% penicillin-streptomycin mixture, and maintained as adherent cultures at 37°C in a humidified atmosphere containing 5% CO2. The culture was free of mycoplasma and murine pathogenic viruses (MA Bioproducts). The BxPC-3 cells are characterized by a wild-type (Wt) K-Ras status and sensitivity to gemcitabine, whereas PANC-1 cells present a K-Ras mutation (mt K-ras) and are resistant to gemcitabine.

### Tumor implantation and *in vivo* tumor growth

Studies were performed on a nude mouse xenograft model, as previously described [31]. After a period of adaptation, implantations were performed on 6-week old nude mice weighing 20–25g at the time of cell inoculation. Human pancreatic adenocarcinoma cells were suspended in serum-free medium, and 50 μl of cell suspension containing 3 × 10^6^ BxPc3 cells or 4 × 10^6^ Panc-1 cells were subcutaneously injected into the flank of the mice. The tumor measurements (i.e. length and width) were completed using a vernier caliper, and tumor volume was calculated using the formula for a prolate ellipsoid: {Length (mm) x [width (mm)]^2^ x 0.5, assuming specific gravity to be one and π to be three.

### Treatments and drugs

Once the tumor volume had reached 100 mm^3^, the mice with the two cell lines were randomized to experimental groups for 28 days of treatment.

Consequently, for BxPC-3 cells, the mice were divided into 7 experimental treatment groups: placebo (P), Gemcitabin (G), Everolimus (E), Gemcitabin + Everolimus (G+E), Gemcitabin + Zoledronic acid (G + AZ), Everolimus + Zoledronic acid (E+AZ) and Gemcitabin + Everolimus + Zoledronic acid (G+E+AZ). For Panc-1 cells, the mice were randomized into only 4 experimental treatment groups: placebo (P), Gemcitabin (G), Everolimus + Zoledronic acid (E+AZ) and Gemcitabin + Everolimus + Zoledronic acid (G+E+AZ).

Gemcitabine (GEMZAR, Eli Lilly, France) was administered by peritoneal injection (PI), twice a week at 100 mg/kg of body weight. Zoledronic Acid (Zometa®, Novartis Pharma, France) was administered once a week by PI at 100μg/kg of body weight, and Everolimus (Afinitor®, Novartis Pharma, France) was administered by gavage at 3 mg/kg of body weight. Treatments were provided and prepared by the pharmacy of Angers University Hospital. Control animals in placebo groups received an equal volume of saline solution to the active molecule [32, 33].

### Analysis process

Experiments were carried out for 28 days. Upon completion of the experiment, intracardiac blood sampling was conducted in anesthetized mice by isoflurane gas. Subsequently, the animals were euthanized, the tumors were excised and weighted, and the tumor volume was measured.

### Histopathological analysis

Following tumor excision, a sample was fixed in 4% neutral buffered formalin, routinely processed and paraffin-embedded. Histopathological analysis was performed using conventional hematoxylin and eosin staining of tissue sections. Evaluation of tumor cell proliferation in primary tumors was performed using Ki67 (DAKO, M7240-MIB France) staining by immunohistochemistry.

### Immunoblot analysis

Protein extracts from tumor tissue samples were prepared by suspending the cells in a cell lysis buffer (Sigma) and protease inhibitor cocktail (Roche). Each extract was prepared as above and an equivalent to 20 μg total protein was separated by SDS-PAGE.

Annexin II Antibody (ab41803, Abcam©, France); hENT1 (antibody LS-C178673, LSBio©, France); AKT (ab8932, Abcam©, France); ERK-p (9101S Cell signaling, France); Raf-1 (ab173539, Abcam©, France); mTOR (ab1093, Abcam©, France); Caspase-3 (ab47131, Abcam©, France). Horseradish peroxidase-conjugated anti-mouse (Promega W402B 28570702©, France) or anti-rabbit IgG (w401B 29303402© Promega, France) was used to detect specific proteins.

Detection of specific proteins was carried out using an enhanced chemiluminescence western blotting kit (Pierce).

We measured the intensity of each band using LAS 4000 software and calculated the relative protein levels normalized to that of the β-actin antibody (A5316-2ML SIGMA ©, France).

### Statistical analysis

We used descriptive statistics (mean, standard deviation (SD), median, minimum and maximum values) and doxplots for comparing distributions of quantitative variables across experimental groups. Differences between groups were assessed using the non-parametric Wilcoxon-Mann-Whitney tests and when the results were significant (p<0.05), the comparison 2 to 2 were assessed using Kruskal-Walis test to compare combined treatments. Significance was set at p ≤ 0.05.

### Statement of translational relevance

Everolimus used as a single agent has not an anti-tumor effect in patients with gemcitabine-refractory metastatic pancreatic adenocarcinoma. This study has shown a new therapeutic approach for treatment of pancreatic adenocarcinoma and the synergic efficacy of zoledronic acid, everolimus and gemcitabine. We would realize a phase I-II study to evaluate the efficacy of combination of these three treatments in pancreatic adenocarcinoma with K-ras mutation and Annexin II overexpression. The aim of this study would be increase objective response to gemcitabine from 5 to 15%. We would test everolimus 5 and 10 mg in first treatment.

## References

[1] Leone N, Voirin N, Roche L, Binder-Foucard F, Woronoff AS, Delafosse P, Remontet L, Bossard N, Uhry Z (2015). [Projection of Cancer Incidence and Mortality in Metropolitan France in 2015]. [Article in French]. http://invs.santepubliquefrance.fr/Publications-et-outils/Rapports-et-syntheses/Maladies-chroniques-et-traumatismes/2015/Projection-de-l-incidence-et-de-la-mortalite-par-cancer-en-France-metropolitaine-en-2015.

[2] Ferlay J, Soerjomataram I, Dikshit R, Eser S, Mathers C, Rebelo M, Parkin DM, Forman D, Bray F (2015). Cancer incidence and mortality worldwide: sources, methods and major patterns in GLOBOCAN 2012. Int J Cancer.

[3] Lepage C, Capocaccia R, Hackl M, Lemmens V, Molina E, Pierannunzio D, Sant M, Trama A, Faivre J, Hackl M, Zielonke N, Oberaigner W, Van Eycken E, EUROCARE-5 Working Group (2015). Survival in patients with primary liver cancer, gallbladder and extrahepatic biliary tract cancer and pancreatic cancer in Europe 1999-2007: results of EUROCARE-5. Eur J Cancer.

[4] Moore MJ, Goldstein D, Hamm J, Figer A, Hecht JR, Gallinger S, Au HJ, Murawa P, Walde D, Wolff RA, Campos D, Lim R, Ding K, National Cancer Institute of Canada Clinical Trials Group (2007). Erlotinib plus gemcitabine compared with gemcitabine alone in patients with advanced pancreatic cancer: a phase III trial of the National Cancer Institute of Canada Clinical Trials Group. J Clin Oncol.

[5] Von Hoff DD, Ervin T, Arena FP, Chiorean EG, Infante J, Moore M, Seay T, Tjulandin SA, Ma WW, Saleh MN, Harris M, Reni M, Dowden S (2013). Increased survival in pancreatic cancer with nab-paclitaxel plus gemcitabine. N Engl J Med.

[6] Neoptolemos JP, Palmer DH, Ghaneh P, Psarelli EE, Valle JW, Halloran CM, Faluyi O, O’Reilly DA, Cunningham D, Wadsley J, Darby S, Meyer T, Gillmore R, European Study Group for Pancreatic Cancer (2017). Comparison of adjuvant gemcitabine and capecitabine with gemcitabine monotherapy in patients with resected pancreatic cancer (ESPAC-4): a multicentre, open-label, randomised, phase 3 trial. Lancet.

[7] Mini E, Nobili S, Caciagli B, Landini I, Mazzei T (2006). Cellular pharmacology of gemcitabine. Ann Oncol.

[8] Binenbaum Y, Na’ara S, Gil Z (2015). Gemcitabine resistance in pancreatic ductal adenocarcinoma. Drug Resist Updat.

[9] Kagawa S, Takano S, Yoshitomi H, Kimura F, Satoh M, Shimizu H, Yoshidome H, Ohtsuka M, Kato A, Furukawa K, Matsushita K, Nomura F, Miyazaki M (2012). Akt/mTOR signaling pathway is crucial for gemcitabine resistance induced by Annexin II in pancreatic cancer cells. J Surg Res.

[10] Tuncyurek P, Mayer JM, Klug F, Dillmann S, Henne-Bruns D, Keller F, Stracke S (2007). Everolimus and mycophenolate mofetil sensitize human pancreatic cancer cells to gemcitabine *in vitro*: a novel adjunct to standard chemotherapy?. Eur Surg Res.

[11] Ryan DP, Hong TS, Bardeesy N (2014). Pancreatic Adenocarcinoma. N Engl J Med.

[12] Neuzillet C, Hammel P, Tijeras-Raballand A, Couvelard A, Raymond E (2013). Targeting the Ras-ERK pathway in pancreatic adenocarcinoma. Cancer Metastasis Rev.

[13] Rajalingam K, Schreck R, Rapp UR, Albert S (2007). Ras oncogenes and their downstream targets. Biochim Biophys Acta.

[14] Tassone P, Tagliaferri P, Viscomi C, Palmieri C, Caraglia M, D’Alessandro A, Galea E, Goel A, Abbruzzese A, Boland CR, Venuta S (2003). Zoledronic acid induces antiproliferative and apoptotic effects in human pancreatic cancer cells *in vitro*. Br J Cancer.

[15] Wei F, Liu Y, Bellail AC, Olson JJ, Sun SY, Lu G, Ding L, Yuan C, Wang G, Hao C (2012). K-Ras mutation-mediated IGF-1-induced feedback ERK activation contributes to the rapalog resistance in pancreatic ductal adenocarcinomas. Cancer Lett.

[16] Wee S, Jagani Z, Xiang KX, Loo A, Dorsch M, Yao YM, Sellers WR, Lengauer C, Stegmeier F (2009). PI3K pathway activation mediates resistance to MEK inhibitors in KRAS mutant cancers. Cancer Res.

[17] Wolpin BM, Hezel AF, Abrams T, Blaszkowsky LS, Meyerhardt JA, Chan JA, Enzinger PC, Allen B, Clark JW, Ryan DP, Fuchs CS (2009). Oral mTOR inhibitor everolimus in patients with gemcitabine-refractory metastatic pancreatic cancer. J Clin Oncol.

[18] Burris HA, Moore MJ, Andersen J, Green MR, Rothenberg ML, Modiano MR, Cripps MC, Portenoy RK, Storniolo AM, Tarassoff P, Nelson R, Dorr FA, Stephens CD, Von Hoff DD (1997). Improvements in survival and clinical benefit with gemcitabine as first-line therapy for patients with advanced pancreas cancer: a randomized trial. J Clin Oncol.

[19] Ma XM, Blenis J (2009). Molecular mechanisms of mTOR-mediated translational control. Nat Rev Mol Cell Biol.

[20] Mendoza MC, Er EE, Blenis J (2011). The Ras-ERK and PI3K-mTOR pathways: cross-talk and compensation. Trends Biochem Sci.

[21] Di Nicolantonio F, Arena S, Tabernero J, Grosso S, Molinari F, Macarulla T, Russo M, Cancelliere C, Zecchin D, Mazzucchelli L, Sasazuki T, Shirasawa S, Geuna M (2010). Deregulation of the PI3K and KRAS signaling pathways in human cancer cells determines their response to everolimus. J Clin Invest.

[22] Carracedo A, Ma L, Teruya-Feldstein J, Rojo F, Salmena L, Alimonti A, Egia A, Sasaki AT, Thomas G, Kozma SC, Papa A, Nardella C, Cantley LC (2008). Inhibition of mTORC1 leads to MAPK pathway activation through a PI3K-dependent feedback loop in human cancer. J Clin Invest.

[23] Schech AJ, Kazi AA, Gilani RA, Brodie AH (2013). Zoledronic acid reverses the epithelial-mesenchymal transition and inhibits self-renewal of breast cancer cells through inactivation of NF-κB. Mol Cancer Ther.

[24] Prabhu L, Mundade R, Korc M, Loehrer PJ, Lu T (2014). Critical role of NF-κB in pancreatic cancer. Oncotarget.

[25] Chung V, McDonough S, Philip PA, Cardin D, Wang-Gillam A, Hui L, Tejani MA, Seery TE, Dy IA, Al Baghdadi T, Hendifar AE, Doyle LA, Lowy AM (2017). Effect of Selumetinib and MK-2206 vs Oxaliplatin and Fluorouracil in Patients With Metastatic Pancreatic Cancer After Prior Therapy: SWOG S1115 Study Randomized Clinical Trial. JAMA Oncol.

[26] Zhao M, Tominaga Y, Ohuchida K, Mizumoto K, Cui L, Kozono S, Fujita H, Maeyama R, Toma H, Tanaka M (2012). Significance of combination therapy of zoledronic acid and gemcitabine on pancreatic cancer. Cancer Sci.

[27] Takano S, Togawa A, Yoshitomi H, Shida T, Kimura F, Shimizu H, Yoshidome H, Ohtsuka M, Kato A, Tomonaga T, Nomura F, Miyazaki M (2008). Annexin II overexpression predicts rapid recurrence after surgery in pancreatic cancer patients undergoing gemcitabine-adjuvant chemotherapy. Ann Surg Oncol.

[28] Jung H, Kim JS, Kim WK, Oh KJ, Kim JM, Lee HJ, Han BS, Kim DS, Seo YS, Lee SC, Park SG, Bae KH (2015). Intracellular annexin A2 regulates NF-κB signaling by binding to the p50 subunit: implications for gemcitabine resistance in pancreatic cancer. Cell Death Dis.

[29] Maréchal R, Mackey JR, Lai R, Demetter P, Peeters M, Polus M, Cass CE, Young J, Salmon I, Devière J, Van Laethem JL (2009). Human equilibrative nucleoside transporter 1 and human concentrative nucleoside transporter 3 predict survival after adjuvant g emcitabine therapy in resected pancreatic adenocarcinoma. Clin Cancer Res.

[30] Javle MM, Shroff RT, Xiong H, Varadhachary GA, Fogelman D, Reddy SA, Davis D, Zhang Y, Wolff RA, Abbruzzese JL (2010). Inhibition of the mammalian target of rapamycin (mTOR) in advanced pancreatic cancer: results of two phase II studies. BMC Cancer.

[31] Schwarz RE, Schwarz MA (2004). *In vivo* therapy of local tumor progression by targeting vascular endothelium with EMAP-II. J Surg Res.

[32] Rubio-Viqueira B, Jimeno A, Cusatis G, Zhang X, Iacobuzio-Donahue C, Karikari C, Shi C, Danenberg K, Danenberg PV, Kuramochi H, Tanaka K, Singh S, Salimi-Moosavi H (2006). An *in vivo* platform for translational drug development in pancreatic cancer. Clin Cancer Res.

[33] Ottewell PD, Mönkkönen H, Jones M, Lefley DV, Coleman RE, Holen I (2008). Antitumor effects of doxorubicin followed by zoledronic acid in a mouse model of breast cancer. J Natl Cancer Inst.

